# Investigating the adaptive coping mechanisms of rewilded elephants: A comparison of behavioural and physiological variables with wild elephants

**DOI:** 10.1371/journal.pone.0348698

**Published:** 2026-07-29

**Authors:** Tenisha Roos, Tamara Eggeling, Marion Garai, Victoria L. Boult, André Ganswindt, Brett Mitchell, Audrey Delsink

**Affiliations:** 1 Elephant Reintegration Trust, Port Alfred, Eastern Cape, South Africa; 2 School of Biological Sciences, University of Reading, Reading, United Kingdom; 3 Mammal Research Institute, Faculty of Natural and Agricultural Sciences, University of Pretoria, Pretoria, South Africa; 4 Humane World for Animals South Africa Trust, Cape Town, Western Cape, South Africa; Tshwane University of Technology, SOUTH AFRICA

## Abstract

Literature on the adaptability and environmental processing abilities of ex-captive (rewilded) African elephants (*Loxodonta africana*) is sparse, emphasising the importance of this research area. By broadening our knowledge on the topic of rewilded elephant behaviour, we can generate a realistic expectation for stakeholders wanting to secure locations for these captive elephants with the aim of rewilding them. We studied 11 African elephants who have been rewilded onto various fenced reserves in South Africa. We measured their adaptability and response to their environment by comparing their behavioural frequencies within four categories and their physiological responses (faecal glucocorticoid metabolite (fGCM) concentrations), to those observed for free-roaming elephants in South Africa. All study animals were grouped into three age/sex categories (Adult females (AF); Adult males (AM); Sub-adult females (SAF)). The results demonstrated that rewilded elephants expressed similar behavioural frequencies to their wild counterparts, except within the “Attentive” category, where both rewilded AMs and SAFs showed significantly lower frequencies than wild elephants. On the contrary, rewilded AFs and SAFs had comparatively higher fGCM concentrations than wild elephants. Through individual comparisons, it was evident that rewilded elephants have adopted unique mechanisms to process environmental stimuli. When the relationship between behavioural and physiological responses was investigated, certain rewilded elephants showed a strong relationship between the two parameters. However, for other elephants, there was no clear correlation between behavioural frequencies and fGCM concentrations. These distinctive differences might largely be influenced by personality traits that will determine whether the elephants respond actively or passively to their environment. This study has shown that rewilded elephants can adapt to wild environments after long-term captivity and that each of them implemented unique ways of coping with both natural and management-related pressures in the different reserves.

## Introduction

African elephants (*Loxodonta* a*fricana*), with their sentience and intelligence, serve as a keystone species, embodying a convergence of intrinsic, socio-cultural, and ecological values [1]. Despite this reverence, they suffer exploitation in multiple ways, such as being captured from the wild to be used in captivity for commercial gain [2–4], poaching for the ivory trade [5], and culling to manage local overpopulation [6], highlighting a global ethical dilemma. Historically, the concept of elephants in captivity was widely accepted by the public, elephant facility managers, and some researchers [7]. In countries such as Asia, it was argued that elephants needed jobs, and they were treated like domestic animals [8] despite their well-being being compromised on multiple levels [2,9–11]. Many studies assessing the well-being of elephants in captivity have shown that they may exhibit a variety of behavioural abnormalities, such as stereotypic behaviour [12–14], aggressive behaviour toward their handlers [15] and conspecifics [16] and increased self-directed touching [17,18] when they are uncomfortable or unable to escape their circumstances. Physiological and neurological impairments due to changes in brain structure after prolonged periods in captivity have also been reported by scientists [19].

In South Africa, approximately 88 African elephants are kept in 22 captive facilities across the country [20]. Most captive elephant groups comprise fewer than 10 individuals [15], signifying that all these groups can be classified as having incomplete social structures due to them lacking the most important family tiers described by Wittemyer et al. 2005 [21]. Synonymous to their wild conspecifics in managed fenced reserves [22], captive elephants may also become ‘challenging’ when housed in incomplete social systems. ‘Challenging’ elephants are often characterised by aggressive behavioural outbursts towards humans or other elephants, and they often gain a reputation for being uncontrollable [23]. Individuals with certain characteristic traits, such as aggression or dominance [24,25] may no longer willingly cooperate with their handlers, possibly as a result of post-traumatic stress disorder (PTSD) caused by their training programmes or during capture [23], conflict between group members [26], or the stress associated with everyday activities and confinement [10,11]. When the individual or group becomes unmanageable, the only solutions proposed are euthanasia [15], translocation to another captive facility, treatment with Gonadotropin hormone-releasing hormone (GnRH) to suppress testosterone induced aggressive behaviour and musth [27,28] in bulls, or release back into the wild (reintegration with the aim of rewilding) [26,29,30].

Some captive elephants may be physically or emotionally compromised, and before these elephants can be reintegrated and ultimately rewilded, they need to be rehabilitated. Rehabilitation involves the treatment of illnesses or injuries, whilst also rebuilding cognitive and physical skills, to increase their chances of survival [31]. Thereafter, the reintegration process can be implemented where the elephants learn to regain autonomy through a systematic reduction in anthropogenic control and involvement [30] in their daily routine. Various elephant reintegration programmes have been established and can vary from a “hard release” where no rehabilitation programme is implemented [29], minimal habituation and rehabilitation [26], to a “soft release” where a full rewilding programme is designed, and comprises of several phases to ensure that the elephants are equipped with the necessary skills to survive in a natural environment [14,32]. When elephants have learned to navigate space and terrain, forage for themselves, and form social groupings, they can be considered “rewilded elephants” [31]. Aside from their general survival, reproduction, and interaction with their wild conspecifics, limited research has been conducted on how rewilded elephants cope in wild systems by processing and responding to environmental changes. This information can provide valuable information on the well-being, as well as the unique differences of these rewilded elephants.

One definition of welfare is the state in which the animal attempts to cope with its environment [ 33–35], whereas the well-being of an animal includes the physical and psychological state of an animal by also including the animal’s ability to express natural behaviours [36]. By assessing elephants’ movement patterns [26,29,30], detailed behavioural frequencies [14,37], as well as physiological responses such as variations in faecal glucocorticoid metabolite (fGCM) concentrations [14], researchers can start to develop a better understanding of how animals perceive their environment. Innovative studies on the well-being of rewilded and wild elephants in Southern Africa include those that have developed concepts such as the “One Well-being” approach [6], which encourages the conservation community to develop more ethical and sustainable conservation and management strategies that benefit humans, animals and the environment. Additional tools published in 2022 [17,37] and 2023 [14,18] utilised detailed behavioural frequencies and physiological parameters to decode the emotional responses of elephants in wild and captive environments. These studies focused on the effects of different variables, such as tourism pressure, reserve characteristics, and elephant population dynamics on the physiological responses (hormone levels) and behavioural frequencies of wild and captive elephants in South Africa.

These researchers acknowledged elephants’ higher-order of intelligence [38–40] by assessing their ability to express their emotions in various ways, such as through detailed behavioural cues indicating when they are experiencing pleasure or discomfort [14,17,37,41]. Additionally, the state of their physiological well-being can be expressed through certain parameters such as fluctuating hormone levels [14,17,42]. Glucocorticoids (GC) are considered metabolic regulators with the function of aiding in preparing the animal to respond to stressors and are therefore often used as proxies of stress responses [43,44]. However, their primary function is energy mobilization, which includes the regulation of carbohydrate metabolism [43]. Thus, researchers should interpret assumed pressure-related alterations in GC concentrations with caution, as these hormones are only one component of a complex suite of physiological and behavioural responses to challenges [43]. During both positive and negative situations, the hypothalamic-pituitary-adrenocortical (HPA) axis is activated, and increased GC secretion occurs [45], facilitating a shift in energy balance to facilitate coping with a challenge [43]. It has been suggested that glucocorticoids should be thought of as mediators of recovery after a high-arousal event, aiding in preparing the body for subsequent demanding situations [46]. Literature has shown that measuring faecal glucocorticoid metabolite (fGCM) concentrations and how they change in elephants has proven to be a successful and non-invasive tool to understand elephants’ response and exposure to pressures and HPA axis activity in an animal [14,37,42,47].

The majority (78%) of elephants who reside in captive facilities in South Africa were captured from the wild [15] for reasons other than conservation [48]. When animals are wild caught, the trauma experienced by the individuals can lead to physiological problems, resulting in ‘chronic stress’ [49]. Long-term stressors can result in changes in the GC regulation, which in turn affects the GC response, whether it is the baseline concentrations, stress-induced concentrations, or the negative feedback system [50]. The baseline concentration of an animal refers to the ‘normal’ level of hormones when the animal excretes when it is not experiencing acute pressures [51]. The Cort-Fitness hypothesis proposed that under prolonged exposure to disturbances, the stress response loses its adaptive function. Thus, an animal is more vulnerable to physiological malfunctions, as an increase in GCs will result in the decline of all fitness metrics [44,52]. Multiple factors can cause changes in GC concentrations in wild elephants [44], such as anthropogenic presence [42,53] management interventions [37], elephant demography [37,42], environmental conditions (season, vegetation, time of day) [42,51,54,55], health-related stressors [56], social structure (55, 57), reproductive status [47,58], etc [44]. Literature has shown that elephants have individual personalities, and that this could also lead to differences in behavioural and physiological expressions [24,25]. Pretorius et al. (2023) confirmed this in their study, which showed the differences in fGCM levels of individual elephants as they transitioned from captivity to roaming free as wild elephants. Interestingly, one sub-adult female in their study had elevated fGCM concentrations in comparison to the other elephants (9 elephants) when she was in captivity, as well as when she was released and roaming free, emphasizing individual reactions to pressures and individual GC levels.

Studies reporting on the adaptability and flexibility of rewilded elephants are few and far between [14,26,30,59,60], especially those that have monitored elephants who have been roaming free for almost a decade. Therefore, we monitored several rewilded elephants in fenced reserves across South Africa who had previously been kept in captivity. Our study aimed to compare the general behavioural and physiological (fGCM concentration) responses of rewilded African elephants to those observed for wild African elephants, to assess their responses in a wild environment. We hypothesised that a) rewilded elephants will show higher frequencies of behaviours that are linked to life in captivity (Conflict and Confrontation, Self-directed touching (SDT) and b) that they will be more aware and wary (higher frequences of Attentive and Vigilant behaviour) of their environment than wild elephants as they were not raised in the wild and may be less habituated to natural pressures [37,41,61]. In addition to their behavioural responses, we hypothesise that c) rewilded elephants will have higher fGCM concentrations (higher HPA axis activity) than their wild counterparts due to their past life experiences [23], d) individual rewilded elephants’ fGCM concentrations will differ from those reported for wild elephants, potentially due to personality [24,62] or different physiological requirements (reproduction status or injuries) [47,58]. Lastly, we aimed to determine whether there is a relationship between behavioural and physiological responses and therefore hypothesise that mean behavioural frequencies will correspond with their mean fGCM concentrations.

## Methods

### Study site

Data were collected on four reserves across South Africa. Each reserve differed in size, bioregion type, elephant population, and tourist density. The rewilded elephants roam in Reserves 1, 2, and 3, whereas the monitored wild elephant population roams in Reserve 4 (Table 1).

**Table 1:** Summary of the characteristics of the four reserves, as well as the total number of elephants roaming in them (GD = Guided drive, SD = Self-drive).

Reserve	Biome	Bioregion	Size (ha)	Province	Elephant population	Wild elephants	Ex-captive elephants	Average tourist vehicle density per day	
								GD	SD
**1**	Fynbos	Eastern Fynbos-Renosterveld and South Strandveld	9 000	Western Cape	13	9	4 (Male: 2 Female: 2)	11-20	1-10
**2**	Savanna	Eastern Kalahari Bushveld	90 000	North West	19	13	6 (Male: 3 Female: 3)	0	1-10
**3**		Between Lowveld and Indian Ocean coastal belt	5 600	KwaZulu-Natal	32	31	1 (Male)	1-10	0
**4**		Lowveld	208 800	Limpopo	± 3607^*^	± 3607^*^ Male: 452 Female: 2992	0	30-50	30-50

*This is an open system bordering Kruger National Park; therefore, the numbers may fluctuate each year depending on the season.

### Study animals

A total of 11 rewilded elephants were included in the study, with each elephant having a unique captive history, origin, age and sex classification, and number of years spent in captivity (Table 2) (AM = Adult male (>20 years old); AF = Adult female (>20 years old); SAF = Sub-adult female (7–15 years old)). The rewilded elephants included in this study were either retired from tourist work due to their unwillingness to cooperate or be handled, conflict with other elephants, increased aggressive behaviour towards handlers in a captive environment, or for ethical considerations.

**Table 2:** Summary of the age and sex classification (estimated date of birth (DOB)), origin, and captive history of the elephants that are included in the study, as well as the reserves they were rewilded on and how many years they were in captivity.

Animal ID	Origin	Captive facility (All have closed)	Captive History	Rewild Reserve	Years in Captivity
**Bully** (AM) (1983)	Wild-caught as an orphan, possibly from KNP (Kruger National Park)	Brian Boswell Circus and Elephants of Eden (EOE)	Circus performer and used for tourism interactions	1	±15 (1993 - 2008)
**Mabitsi** (AM) (1988)	Wild caught as calves during the KNP culling operations (1993)	EOE	Used for tourism interactions		±15 (1993 - 2008)
**Bonnie** (AF) (1991)		Brian Boswell Circus	Circus performer		± 17 (1993 - 2010)
**Thembile** (AF) (1991)		EOE	Used for tourism interactions		±17 (1993 - 2010)
**Mana** (AM) (1985)	Wild caught as calves during a culling operation in Zimbabwe (1988)	Pilanesberg Elephant Back Safaris [63]	Used for Elephant Back Safaris (EBS) and interactions	2	±27 (1988–2015)
**Sharu** (AM) (1985)					
**Michael** (AM) (1985)					
**Chikwenya** (AF) (1985)					
**Tidimalo** (SAF) (2009)	Born in a captive facility (Chikwenya’s offspring)		In training for EBS		± 6 (2009–2015)
**Ngwedi** (SAF) (2012)			Used for tourism interactions		± 3 (2012 - 2015)
**Gobisa** (AM) (1984)	Wild caught during culling operation in Zimbabwe (1980s)	Waterberg Elephant Back Safaris (WEBS)	Used for EBS and interactions	3	± 24 (1986–2010)

The rewilded elephants in Reserve 1 were free-roaming with a small herd of 13 wild elephants. Bonnie (AF) and Thembile (AF) both had calves in 2011 and 2012. The two rewilded adult females formed an independent herd and formed part of a fission-fusion system with the wild herd and rewilded bulls. Bully (AM) and Mabitsi (AM) are the only adult bulls in Reserve 1 and roam as lone bulls, associating with each other, the wild, and the rewilded herds. Both these rewilded bulls were treated with Gonadotropin-Releasing Hormone (GnRH) vaccine biannually to suppress testosterone production, which leads to a lack of musth cycles and decreased testosterone-related aggression [27,64]. The administration of this vaccine ceased in 2022. In Reserve 2, Chikwenya (AF) is the only adult female in the rewilded herd and gave birth to a calf in 2019. Tidimalo (SAF) and Ngwedi (SAF) also gave birth to calves in 2019 and 2021 respectively, which means that calves were always present in Reserve 2, throughout the study period. The rewilded herd roamed as a small herd, whilst also associating with a wild breeding herd of seven elephants. Mana (AM) and Sharu (AM) roamed as independent bulls and associated with each other, the rewilded and wild breeding herds. Michael (AM) and Chikwenya (AF) formed a unique bond in captivity, and after their reintegration, Michael (AM) never dissociated from the rewilded herd, unlike the other rewilded bulls. The latter was confirmed by reserve staff and researchers who conducted regular monitoring sessions in the years following their reintegration. Gobisa (AM), roaming in Reserve 3, is the only rewilded bull on the property and forms part of the wild herd, roaming as a lone bull, with other bulls, and with the breeding herds. Gobisa was vaccinated once a year with the GnRH vaccine from 2013 to 2019.

### Reintegration histories of the rewilded elephants

This study had no control over the reintegration programmes that were previously implemented for each of the ex-captive elephants who were monitored over the study period. Each reserve employed unique methods to reintegrate the elephants when they were translocated from their respective captive facilities (Table 3).

**Table 3:** Summary of the reintegration process that each rewilded elephant was exposed to, as well as the number of years they have since been roaming free (beginning to end of data collection).

Reserve	Animal ID	Reintegration process	Years free
1	Bully	‘Hard release’: Bully and Mabitsi did not know each other before their release on Reserve 1. They were kept separate in adjacent bomas (Electric fenced, open-aired temporary holding area) after being translocated to the reserve. Thereafter, they formed a relationship across the dividing fence. After two weeks, they were housed together in one boma. They were released together from the boma without any other form of human intervention.	12-14 years
	Mabitsi		
	Bonnie	‘Hard release’: There was no formal reintegration program. Bonnie and Thembile were put together in a 1-hectare boma on Reserve 1, where they formed a bond. They were both released 48 hours after being put into the boma and shortly joined up with Bully and Mabitsi on their own.	10-14 years
		Thembile	
2	Mana	‘Soft release’: A facilitated reintegration program was employed that gradually habituated the herd to full wild living over a 6–12-month period until the elephants showed signs of being equipped to survive without further human intervention.	4-9 years
	Sharu		
	Michael		
	Chikwenya		
	Tidimalo		
	Ngwedi		
3	Gobisa	‘Hard release’: Released onto the reserve without any reintegration process.	9-14 years

### Data collection

Behavioural data (10-minute video recordings (focal samples)) [65] and physiological data (faecal samples were collected over approximately 5 years (March 2019-July 2024). The elephant populations on each of the reserves were monitored on five to eight separate occasions, with each session consisting of approximately 21 observation days (one session in Reserve 2 and Reserve 1 lasted for more than 21 days). The goal was to collect an equal number of focal (10-minute video recordings) and faecal samples during wet and dry seasons; however, on some reserves, this was not always achieved due to challenges such as elephant accessibility, either due to their location or roads being closed due to weather conditions (Table 4). The researcher monitored the elephants on each reserve for a minimum of four hours and a maximum of eight hours a day. As the study animals are free-roaming elephants, the focal and faecal sample collection relied heavily on the vegetation type that the elephants were roaming in and how far the elephants were from the road. The focal samples were recorded as soon as the elephant (head and body) became visible. When an elephant defecated, the researcher needed to wait for the specific individual to move away so that the sample could be safely collected. This means that we could not standardise at what time the focal and faecal samples were collected each day. A total of 275 focal samples and 264 faecal samples were processed after the completion of the entire data collection period. Due to the characteristics of Reserve 4 (free-roaming elephants open to KNP) (Table 1), it was extremely difficult to ensure that we collected more than one focal sample per specific elephant. Therefore, the ID categories named wild AF, wild AM, and wild SAF consisted of multiple wild individuals combined that fit into the three groups.

**Table 4:** Summary of the observation sessions (period) allocated to the reserves during wet and dry rainfall seasons, as well as the number of samples (focal; faecal) collected within each session.

***Reserve***	***Season***	***Dry***			***Wet***					**Total**
***1***	***Period***	**Jan 2020**	**Jan-Feb 2021**	**Mar-May 2024**	**Jun-Jul 2020**	**Sept-Oct 2021**	**Aug-Oct 2023**	**Jun-Jul 2024**		
	***Bonnie (AF)***	*5; 3*	*2; 0*	*0; 2*	*3; 1*	*9; 2*	*1; 3*	*0; 1*		*20; 12*
	***Thembile (AF)***	*3; 0*	*3; 0*	*1; 0*	*4; 1*	*3; 1*	*6; 1*	*0; 1*		*20; 4*
	***Bully (AM)***	*4; 1*	*1; 0*	*0; 2*	*6; 3*	*7; 2*	*2; 5*	*0; 0*		*20; 13*
	***Mabitsi (AM)***	*3; 4*	*1; 0*	*0; 4*	*6; 3*	*8; 1*	*2; 5*	*0; 3*		*20; 20*
	***Total***	***15; 8***	***7; 0***	***1; 8***	***19; 8***	***27; 6***	***11; 14***	***0; 5***		***80; 49***
***Reserve***	***Season***	***Dry***					***Wet***			**Total**
***2***	***Period***	**May-June 2019**	**Aug-Oct 2020**	**May 2022**	**Jul 2023**	**Apr-May 2024**	**Jan 2021**	**Nov 2021**	**Feb 2023**	
	***Chikwenya (AF)***	*0; 1*	*6; 4*	*3; 4*	*0; 3*	*0; 0*	*6; 8*	*4; 2*	*1; 0*	*20; 22*
	***Michael (AM)***	*1; 6*	*5; 14*	*3; 3*	*0; 0*	*0; 0*	*6; 7*	*3; 0*	*0; 2*	*18; 32*
	***Mana (AM)***	*0; 14*	*2; 8*	*6; 3*	*1; 0*	*0; 4*	*3; 6*	*4; 0*	*4; 3*	*20; 38*
	***Sharu (AM)***	*3; 6*	*0; 1*	*6; 4*	*3; 2*	*0; 2*	*1; 2*	*3; 3*	*4; 1*	*20; 21*
	***Tidimalo (SAF)***	*0; 1*	*3; 9*	*3; 1*	*4; 1*	*0; 0*	*4; 4*	*4; 2*	*1; 0*	*19; 18*
	***Ngwedi (SAF)***	*0; 0*	*4; 3*	*3; 2*	*5; 2*	*0; 0*	*5; 5*	*2; 1*	*1; 1*	*20; 14*
	***Total***	***4; 28***	***20; 39***	***24; 17***	***13; 8***	***0; 6***	***25; 32***	***20; 8***	***11; 7***	***117; 145***
***Reserve***	***Season***	***Dry***			***Wet***					***Total***
***3***	***Period***	***Aug 2019***	***Sept 2022***	***July 2023***	***Dec 2020***	***Oct 2021***	***Feb 2024***			
	***Gobisa (AM)***	*1; 2*	*2; 2*	*4; 3*	*2; 1*	*2; 2*	*8; 3*			***19; 13***
***Reserve***	***Season***	***Dry***		***Wet***						***Total***
***4***	***Period***	**Apr 2019**	***Aug-Sept 2021***	***Nov-Dec 2019***	***Nov 2020***	***Mar-Apr 2022***				
	***Wild AF***	*0; 3*	*10; 0*	*2; 9*	*3; 12*	*5; 0*				*20; 24*
	***Wild AM***	*1; 8*	*7; 0*	*4; 7*	*1; 3*	*7; 0*				*20; 18*
	***Wild SAF***	*2; 4*	*7; 2*	*0; 2*	*5; 4*	*5; 3*				*19; 15*
	***Total***	***3; 15***	***24; 2***	***6; 18***	***9; 19***	***17; 3***				***59; 57***

### Behavioural data collection protocol

Prior to the study, a period was allowed to enable the elephants to get habituated to the research vehicle. The researcher implemented each reserve’s elephant approach protocol and aimed not to disturb the elephants during the data collection periods. If the elephants chose to move or feed toward the vehicle, the observer would remain in the initial position. The behavioural data collection consisted of acquiring 10-minute video recordings (focal samples). The 10-minute video recordings were processed following the completion of the fieldwork.

### Behavioural data processing

Although a standardised video processing protocol was followed, the focal samples in this study were all processed by one observer to minimise any observer discrepancies that may occur with multiple observers. Focal samples were only processed where the elephants were not disturbed and not interacting with the research vehicle. The researcher kept a neutral presence by maintaining a minimum distance of 30m from the focal elephant. For example, if the elephant/s decided to investigate the vehicle, the researcher would only start recording when the elephant/s had moved away and settled back into a natural activity such as feeding, or drinking, etc. The video processing consisted of tallying the total frequencies of the detailed behaviours that occurred within each 10-minute video recording. The detailed behaviours exhibited by the focal elephants were identified by consulting an ethogram (Table 5) that was compiled based on existing literature [17,18,37,41,61]. Thereafter, the detailed behaviours that fell into specific behavioural categories were tallied to summarise the total frequencies within each behavioural category. The four behavioural categories were defined as: Self-directed touching [17], Vigilance, Attentive, and Conflict & Confrontation behaviour

**Table 5:** Description of the behavioural categories and the detailed behaviours (and the codes) that comprised each of them.

Behavioural category	Behaviour	Code	Description
**Self-directed touching (SDT)** All the behaviours that occur and did not form part of body care behaviour such as swatting flies, mud bathing, scratching body etc.	Touching/ brushing face	TEF	The trunk tip touches/ brushes the face of the elephant
	Rubbing or touching eye	REYE	The tip of the elephant’s trunk rubs the eye
	Rubbing or touching ear	REAR	The elephant rubs their ear with the tip of their trunk
	Touching/ brushing leg or foot	TLF	The tip of the trunk swings/touches the elephant’s leg or foot
	Touching mouth	TMO	Tip of the trunk touches their mouth or is placed in their mouth or pulled down while biting the tip
	Trunk on head + Trunk push head	TOH	The trunk lies flat on the elephant’s head, or the tip of the trunk pushes against the forehead
	Trunk curl	TCR	The tip of the trunk is curled inwards to form a ball
	Trunk twist and twirl	TTT	Trunk twisted and twirled on itself or to the sides
	Trunk to trunk	TTR	Tip of trunk touches another part of the trunk
	Trunk between tusk and face	TTF	Tip of trunk is held between the tusk and the face
	Trunk over tusk	TOT	Trunk is draped over the tusk, not related to resting the trunk
	Trunk to body	TBO	Tip of trunk touches any part of the elephant’s body
	Hanging trunk rotate left to right	HTR	Tip of trunk is rotated left and right
	Trunk to tusk / tusk socket	TTS	The elephant touched the tusk socket with the tip of the trunk
**Vigilance** Any behaviour occurring in response to an external stimulus	Ears are spread	EAS	Both ears are spread and kept still
	Head held high or standing tall	HHH	Head is held above the shoulders and both ears are spread out
	Walking warily or fast	WAR	Walks with tail up, and ears spread
	Lifting foot and swinging	FSW	Stands still while the front- or back foot is swung back and forth
	Tail pulled to side	TPS	The tail is lifted and pulled to the side
**Attentive** Any behaviour occurring when the elephant is trying to gain information of the environment	J-Trunk (Smelling air)	SMA	Tip of trunk is curled towards the direction the elephant is smelling (Excluding vegetation)
	Smelling dung or urine	SDU	Top of trunk is pointed towards dung or urine
	Freezing	SPL	Stands still for a few seconds to listen, look, or feel vibrations through the ground
	Lift trunk to smell	LTS – Air	Tip of trunk is lifted upwards above the mouth line
	Explore with trunk – object	EWT-OBJ	Tip of trunk touches object
	Explore with foot	EWF	Foot is used to touch an object
	Lifting/ Picking up item	LIO	Tip of trunk picks up or lifts an item (not related to feeding)
**Conflict & Confrontation** When the elephants are giving a warning sign of them being either uncomfortable or irritated	Head shake	HES	Head is shaken from side to side in an abrupt manner
	Throw item	THI	Object is thrown in a specific direction
	Trunk swing/ swish	TRS	Trunk is flicked forward

At the beginning of each focal sample, different variables (general activity, distance from vehicle, elephant identification (ID), and rainfall season) were recorded to provide a full overview of the environmental conditions during each video recording.

### Physiological data collection protocol

#### Faecal sample collection.

During the field observation sessions, when the monitored elephants were seen defecating, the researcher opportunistically collected 50-100g of faecal material from the centre of one or more dung boli, mixed the contents, and placed them in plastic bottles less than two hours post-defecation. Faecal samples were kept on ice until they could be frozen at −18°C when the researcher returned to the research base each day. The samples were kept frozen until further processing at the Endocrine Research Laboratory, University of Pretoria, South Africa. Factors such as time of defecation, time of collection, elephant ID (where possible), age and sex, as well as the season during which the sample was collected, were recorded in the field for each faecal sample.

#### Faecal steroid extraction and fGCM quantification.

Faecal samples were lyophilized, pulverized and sifted using a nylon mesh strainer to remove fibrous material [58]. Between 0.050–0.055 g of the faecal powder was then extracted with 80% ethanol in water (3 ml). The suspensions were vortexed for 15 min and subsequently centrifuged at 1500 *g* for 10 min. The supernatants formed were transferred into microcentrifuge tubes and stored at −20°C until analysis. The resulting extracts were measured for immunoreactive fGCM concentrations using an 11-oxoetiocholanolone enzyme immunoassay (EIA), detecting fGCMs with a 5*β*-3*α*-ol-11-one structure. This EIA has been shown to reliably detect adrenocortical function in African elephants [47]. Detailed assay characteristics, including full descriptions of the assay components and antibody cross-reactivities, are provided by Möstl and colleagues [66]. Sensitivity (at 90% binding) of the assay was 1.5 ng/g dry weight (DW). Intra-assay coefficients of variation (CV) determined by repeated measurements of high and low value quality controls were 4.87% and 6.60%. Inter-assay CV determined by repeated measurements of high and low value quality controls were 14.76% and 15.73%. Serial dilutions of faecal extracts gave displacement curves that were parallel to the respective standard curve with a relative variation of the slope of the trend lines *<* 3%. All steroid concentrations are expressed per mass of faecal DW matter. All analyses were conducted at the Endocrine Research Laboratory, University of Pretoria with assay procedures following [67].

### Statistical analysis

We used R version 4.4.2 [68] to analyse data collected for this study. Before analysis, elephants were divided into three groups based on age and sex (Adult females (AFs), Adult males (AMs), and Sub-adult females (SAFs)). When comparing wild to rewilded elephants, we only compared individuals within these groups to remove any influence of age and sex on behavioural and physiological rates. When the detailed behavioural frequencies were investigated, only behaviours that exhibited more than 4% of the total frequencies of behaviours expressed within each of the three groups (AF, AM, and SAF) were included in the analysis.

To compare the frequency of behavioural categories and detailed behaviours between wild and rewilded elephants, we first used a Mann-Whitney U test. Subsequently, we used a Dunnett’s test to explore which of the rewilded individuals differed significantly from the wild baseline. In this formal analysis, the individual rewilded elephants were not compared to one another, only to the wild elephants within the different age and sex classes.

We used a general linear model to examine the influence of captivity status on *fGCM* levels between wild and ex-captive elephants whilst also controlling for season (known to influence fGCM [69]). A Dunnett’s test was again used to identify which rewilded individuals differed significantly from the wild baseline. As a result of limited data points (four) for Thembile, she was removed from this analysis.

To further increase our understanding of the factors that may have contributed towards the potential changes in fGCM concentrations of wild and rewilded elephants, samples collected for fGCM quantification at different times of the day (morning: 05:00–11:59; afternoon: 12:00–19:00), dry and wet seasons, time and captivity, and years roaming free were compared. For the ‘time of defecation’, and ‘seasonal’ effect, a Mann-Whitney U test was used, and for parameters with more than three factors (Years in captivity), a Kruskal–Wallis test with a Dunn’s test (Bonferroni correction) for the post-hoc comparisons was used. All these comparisons were made within the AF, AM, and SAF age and sex categories and were separate for wild and rewilded elephants. For the wild and rewilded SAF, no formal statistical analysis was run when testing for the influence of ‘time of defecation’ as only four samples were collected in the morning for both groups. For the wild AFs, no formal analysis could be conducted for seasonal effect, as only three samples were collected during the dry season.

### Ethical statement and permits

This project was approved by the Research Ethics Committee of the University of Pretoria permit. AEC Reference No.: REC254−19. Permission under Section 20 of the Animal Diseases Act, 1984 (act no 35 of 1984) to perform the research was provided by the Department of Agriculture, Forestry and Fisheries of the Republic of South Africa. No authority permission was required for field access, as all the research was conducted on private land and not government land.

## Results

### A behavioural comparison of rewilded and wild elephants

During this study, a total of 275 (rewilded = 216; wild elephants = 59) 10-minute focal samples (≈ 46 hours) were analysed for which a total behavioural frequency of 1215 (rewilded = 892; wild elephants = 323) was reported within the Attentive-, 81 (rewilded = 61; wild elephants = 20) within the Conflict & Confrontation-, 352 (rewilded = 283; wild elephants = 69) within the Vigilance-, and a total of 1206 (rewilded = 915; wild elephants = 291) within the Self-directed touching (SDT) behavioural category for wild and rewilded elephants combined.

### Rewilded versus wild elephants

Wild AMs (Mann-Whitney U: W = 646, p = 0.001) and SAFs (Mann-Whitney U: W = 211, p = 0.008) showed significantly higher frequencies of Attentive behaviours than their rewilded counterparts (Table 6), while there was no significant difference for AFs. Further, no significant differences were found between wild and rewilded elephants in any of the age/sex class for Conflict & Confrontation, Vigilance, and SDT behavioural categories (Table 6).

**Table 6:** The mean frequencies (per 10-min) ± SE that the wild – and rewilded elephants exhibited within each behavioural category within each of the three age and sex groups (AF, AM, and SAF). N = number of focal samples. P-values in bold indicate statistical significance.

		Adult females		Adult males		Sub-adult females	
		Wild (N = 20)	Rewilded (N = 60)	Wild (N = 20)	Rewilded (N = 117)	Wild (N = 39)	Rewilded (N = 19)
**Attentive**	Mean	2,200	4,300	5,150	3,274	5,842	2,231
	SE	0,551	0,644	0,877	0,438	1,018	0,420
	Median	2,000	3,000	5,500	2,000	6,000	2,000
	Range	8,000	25,000	13,000	39,000	13,000	11,000
	p-value	0.154		**0.001**		**0.008**	
**Conflict & Confrontation**	Mean	0,100	0,317	0,100	0,179	0,842	0,538
	SE	0,069	0,142	0,069	0,098	0,414	0,207
	Median	0,000	0,000	0,000	0,000	0,000	0,000
	Range	1,000	8,000	1,000	11,000	7,000	7,000
	p-value	0.372		0.857		0.539	
**Vigilance**	Mean	0,500	1,100	0,950	1,051	2,105	2,410
	SE	0,199	0,234	0,285	0,157	0,567	0,455
	Median	0,000	1,000	1,000	0,000	1,000	1,000
	Range	3,000	12,000	5,000	9,000	7,000	11,000
	p-value	0.083		0.666		0.766	
**Self-directed touching**	Mean	2,500	5,233	3,200	2,393	9,316	8,231
	SE	0,635	0,925	0,823	0,330	2,220	2,050
	Median	1,500	3,000	2,000	1,000	8,000	4,000
	Range	8,000	38,000	12,000	22,000	32,000	67,000
	p-value	0.052		0.404		0.387	

Considering detailed behaviours, significantly higher frequencies of the “Freezing” (Mann-Whitney U: W = 5407, p = 0.038) behaviour and lower frequencies of the “Rubbing ear” (Mann-Whitney U: W = 4892, p < 0.001) behaviour were noted for the rewilded AFs than for the wild AFs (S1 Table). Wild AMs showed significantly higher frequencies (Mann-Whitney U: W = 5407, p = 0.038) of the “Freezing” behaviour than that observed for the rewilded AMs. The wild SAFs showed significantly higher frequencies (Mann-Whitney U: W = 4892, p < 0.001) of the “Rubbing Ear” behaviour than the rewilded SAFs (S1 Table).

### Individual differences from wild elephants

**Fig 1:**
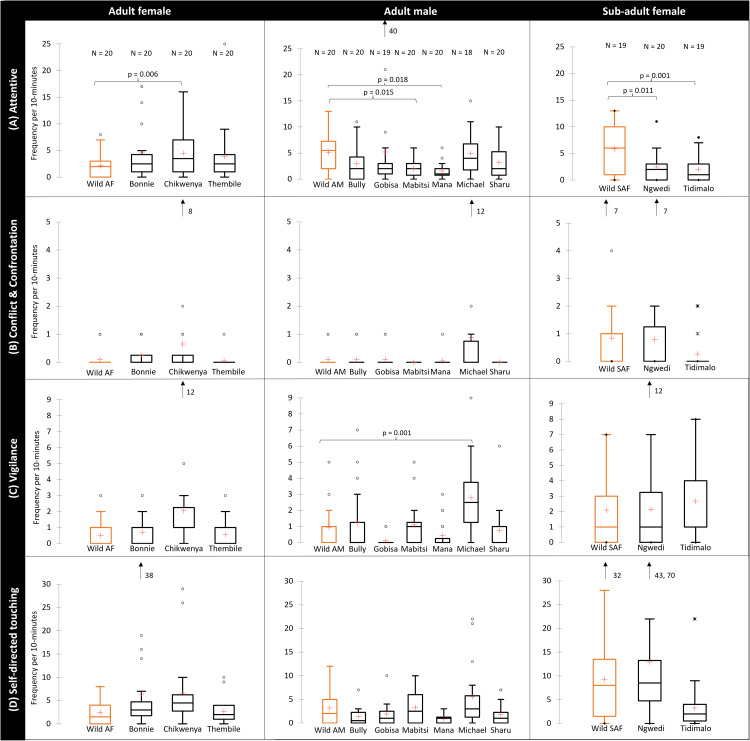
The frequency (per 10-min) of the detailed behaviours that were expressed by the rewilded and wild AFs, AMs and SAF elephants within the (A) Attentive, (B) Conflict & Confrontation, (C) Vigilance, and (D) Self-directed touching behavioural categories. The crosses represent the means, whereas the central horizontal bars are the medians. The first and third quartiles are the lower and upper limits of the box, respectively. The whiskers represent the minimum and maximum datapoints. The points above the whiskers’ upper and lower bounds may be considered outliers. The values with arrows above the y-axes indicate the outlier datapoints that fell outside the scale (N = number of focal samples per elephant). P-values as per Dunnett’s test.

Chikwenya (AF) exhibited significantly higher frequencies (p = 0.006) of Attentive behaviour than the wild AF baseline (Fig 1A). Within the adult male category, Mabitsi (AM) (p = 0.015) and Mana (AM) (p = 0.018) showed significantly lower frequencies of Attentive behaviour than their wild counterparts (Fig 1A). It was noted that Michael (AM) was significantly (p = 0.001) more Vigilant than what was reported for the wild AM baseline. Furthermore, both Ngwedi (SAF) (p = 0.011) and Tidimalo (SAF) (p = 0.001) showed lower frequencies of Attentive behaviour than that shown by the wild SAFs.

**Fig 2:**
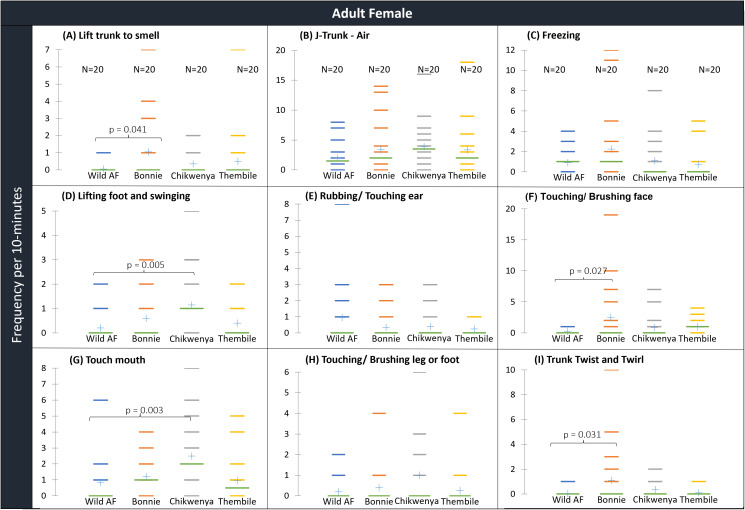
The frequency (per 10-min) of the detailed behaviours (A-I) that were expressed by the rewilded and wild adult female elephants within the behavioural categories. The crosses represent the means, whereas the central horizontal green bars are the medians. (N = number of focal samples per elephant). P-values as per Dunnett’s test.

In terms of detailed behaviours, Bonnie (AF) showed significantly higher frequencies of the “Lift trunk to smell” (p = 0.041), “Touching/Brushing face” (p = 0.027), and “Trunk Twist and Twirl” (p = 0.031) behaviour than those observed for the wild AFs (Figs 2A and F). Chikwenya (AF) showed significantly higher frequencies of the “Lifting foot and swinging behaviour” (p = 0.005) (Fig 2D) and “Touch mouth” (p = 0.003) (Figs 2D and G) behaviour than the wild AFs.

**Fig 3:**
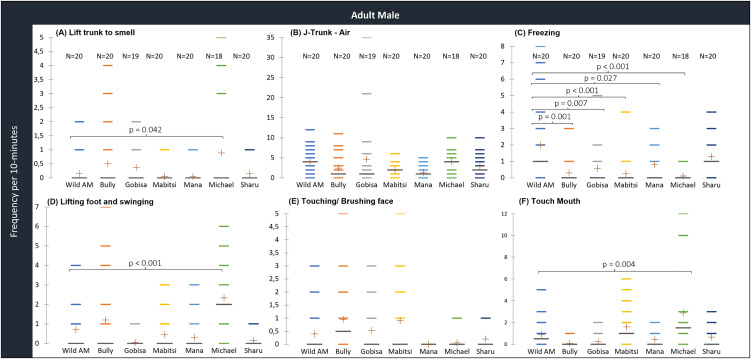
The frequency (per 10-min) of the detailed behaviours (A-F) that were expressed by the rewilded and wild adult male elephants within the behavioural categories. The crosses represent the means, whereas the central horizontal dark blue bars are the medians. (N = number of focal samples per elephant). P-values as per Dunnett’s test.

Michael (AM) showed significantly higher frequencies of the “Lift trunk to smell” (p = 0.042), “Lifting foot and swinging” (p < 0.001), and “Touch Mouth” (p = 0.004) and lower frequencies of the “Freezing” (p < 0.001) behaviour than the wild AMs (Figs 3A, C, D and F). Bully (AM) (p = 0.001), Gobisa (AM) (p = 0.007), Mabitsi (AM) (p < 0.001), and Mana (AM) (p = 0.027) showed significantly lower frequencies of the “Freezing” behaviour than their wild counterparts (Fig 3C).

**Fig 4:**
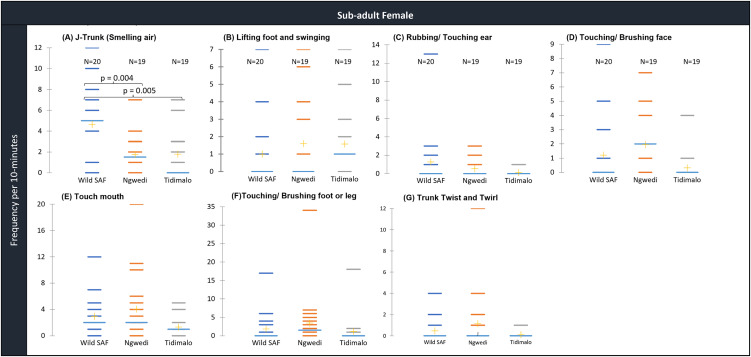
The frequency (per 10-min) of the detailed behaviours (A-G) that were expressed by the rewilded and wild SAF elephants within the behavioural categories. The crosses represent the means, whereas the central horizontal light blue bars are the medians. (N = number of focal samples per elephant). P-values as per Dunnett’s test.

The wild SAF showed significantly higher frequencies of the “J-Trunk (Smelling air)” behaviour than both Tidimalo (SAF) (p = 0.005) and Ngwedi (SAF) (p = 0.004) (Fig 4).

### Physiological comparison of wild and rewilded elephants

#### Faecal Glucocorticoid Metabolite Concentrations (fGCM).

**Fig 5:**
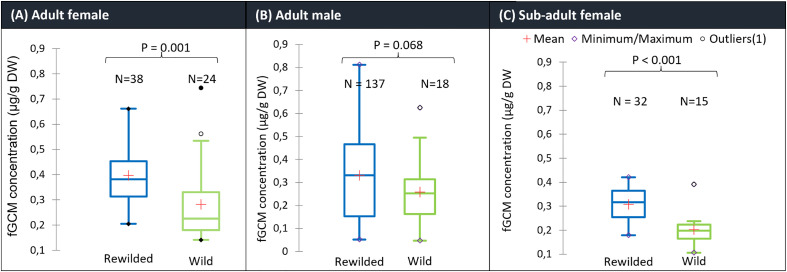
Box plot illustrating the faecal glucocorticoid metabolite concentration (μg/g DW) of rewilded and wild (A) Adult females, (B) Adult males, and (C) Sub-adult females. The crosses represent means, whereas the central horizontal bars show medians. The first and third quartiles are the lower and upper limits of the box, respectively. The length of the whiskers indicates 1.5 times the interquartile range. The points above the upper limits of the whiskers are outliers (hollow circles). Minimum values and maximum values corresponded to the uppermost outlier and indicated as purple diamonds (N = number of faecal samples). P-values as per GLM.

Rewilded adult (GLM: t-value = −3.41, p-value = 0.001) and sub-adult (GLM: t-value = −5.08, p-value < 0.001) females exhibited significantly higher fGCM concentrations than their wild counterparts (Fig 5A and C). We noted a 51.72% difference in overall median fGCM concentrations for wild AFs (median = 0.225 μg/g DW) compared to rewilded AFs (median = 0.382 μg/g DW). We also noted a 45.44% difference between the overall median fGCM concentrations of the wild- (median = 0.199 μg/g DW) and rewilded SAFs (median = 0.316 μg/g DW) (Fig 5C). We found no significant difference in fGCM concentrations for wild versus rewilded males, but did find a significant influence of season (GLM: t-value = 3.25, p-value = 0.001) (Fig 5B).

#### Individual differences from wild elephants.

**Fig 6:**
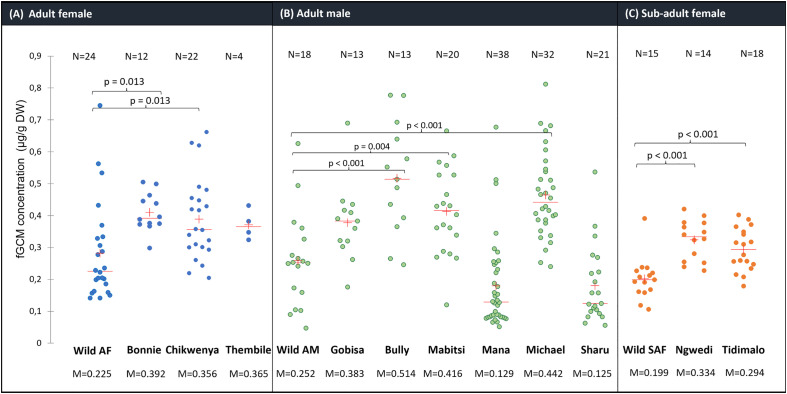
Scatter plot illustrating the faecal glucocorticoid metabolite concentration (μg/g DW) of the individual elephants within the (A) Adult females, (B) Adult Males, (C) Sub-adult females age and sex categories.The crosses represent means, whereas the central red horizontal bars show medians (N = number of faecal samples; M = Median concentrations).

When we compared the fGCM levels of the individual rewilded elephants to the concentrations reported for the wild elephants, Bonnie (AF) (median = 0.392 μg/g DW; p = 0.013; 54.13% difference), and Chikwenya (AF) (median = 0.356 μg/g DW; p = 0.013; 45.10% difference) had significantly higher fGCM concentrations than the wild AFs (overall median = 0.225 μg/g DW)(Fig 6A; Table 7).

**Table 7:** The fGCM concentrations of the wild – and rewilded elephants. N = number of focal samples.

Age and sex	Elephant ID	Median	Mean	Standard deviation	Range	Variation coefficient	CI (Lower bound (95%))	CI (Upper bound (95%))	N
**Adult female**	Wild AF	0.225	0.282	0.150	0.604	0.530	0.218	0.347	24
	Bonnie	0.392	0.411	0.058	0.207	0.142	0.372	0.449	12
	Chikwenya	0.356	0.389	0.127	0.457	0.327	0.331	0.447	22
	Thembile	0.365	0.372	0.040	0.107	0.109	0.297	0.446	4
**Adult male**	Wild AM	0.252	0.259	0.141	0.578	0.546	0.187	0.331	18
	Gobisa	0.383	0.378	0.116	0.513	0.306	0.305	0.451	13
	Bully	0.514	0.517	0.168	0.532	0.326	0.411	0.623	13
	Mabitsi	0.416	0.413	0.131	0.545	0.317	0.350	0.476	20
	Mana	0.129	0.180	0.137	0.625	0.766	0.134	0.225	38
	Michael	0.442	0.465	0.133	0.572	0.286	0.416	0.514	32
	Sharu	0.125	0.181	0.118	0.481	0.652	0.126	0.235	21
**Sub-adult female**	Wild SAF	0.199	0.202	0.063	0.285	0.314	0.166	0.238	15
	Ngwedi	0.334	0.324	0.061	0.193	0.187	0.288	0.360	14
	Tidimalo	0.294	0.294	0.065	0.223	0.220	0.261	0.327	18

Within the adult male category, Bully (median = 0.514 μg/g DW; p < 0.001; 68.41% difference), Mabitsi (median = 0.416 μg/g DW; p = 0.004; 49.10% difference), and Michael (median = 0.442 μg/g DW; p < 0.001; 54.76% difference) had significantly higher fGCM levels than the wild AMs (overall median = 0.252 μg/g DW) (Fig 6B; Table 7). Both Ngwedi (median = 0.334 μg/g DW; p < 0.001); 50.66% difference) and Tidimalo (median = 0.294 μg/g DW; p < 0.001; 38.54% difference) had higher fGCM concentrations than the wild SAFs (overall median = 0.199 μg/g DW) (Fig 6C; Table 7).

#### Other parameters influencing the fGCM concentrations of wild and rewilded elephants.

**Fig 7:**
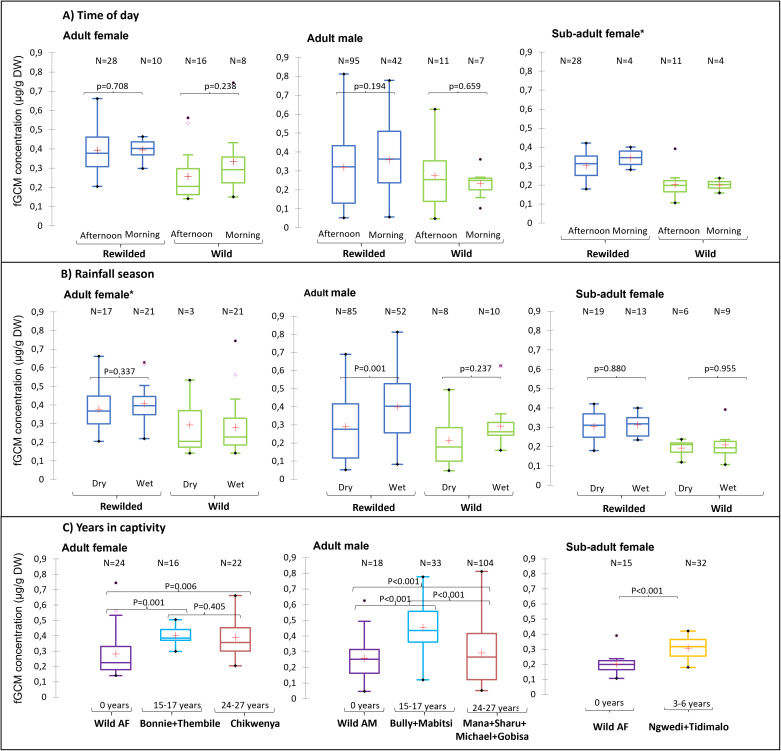
Box plot illustrating the faecal glucocorticoid metabolite concentration (μg/g DW) of rewilded and wild (A) Time of day, (B) Season, and (C) Years in captivity. The crosses represent means, whereas the central horizontal bars show medians. The first and third quartiles are the lower and upper limits of the box, respectively. The length of the whiskers indicates 1.5 times the interquartile range. The points above the upper limits of the whiskers are outliers (hollow, purple circles). Minimum values and maximum values corresponded to the uppermost outlier and are indicated as black diamonds (N = number of faecal samples). *No statistical test was run due to the limited number of samples (N = number of faecal samples).

The time at which the faecal samples for fGCM quantification were collected had no significant effect on the fGCM concentrations of rewilded (p = 0.708) and wild (p = 0.238) AFs, rewilded (p = 0.194) and wild (p = 0.659) AMs (Fig 7a). When the effect of the rainfall season was analysed, no significant effect was observed for the rewilded (p = 0.337) AFs (Fig 7b). Interestingly, the rewilded AMs had significantly (p = 0.001) higher fGCM concentrations during the wet season; however, no significant (p = 0.24) seasonal effect was reported for the wild AMs (Fig 7b). We reported no significant seasonal effect for the rewilded (p = 0.88) and wild (p = 0.96) SAFs (Fig 7b). Furthermore, the AF elephants who have never been in captivity (wild), had significantly lower fGCM concentration than those who have been in captivity for 15–17 years (p = 0.001) and 24–27 years (p = 0.006)(Fig 7c). The AM elephants who had never been in captivity had significantly lower fGCM concentrations than those who had spent 15–17 (p < 0.001) years and 24–27 years (p < 0.001) in captivity. Furthermore, the AMs who spent 15–17 years in captivity had significantly (p < 0.001) higher fGCM concentrations than those who were captive for 24–27 years Fig 7c).

#### Relationship between behavioural frequencies and fGCM concentrations.

**Fig 8:**
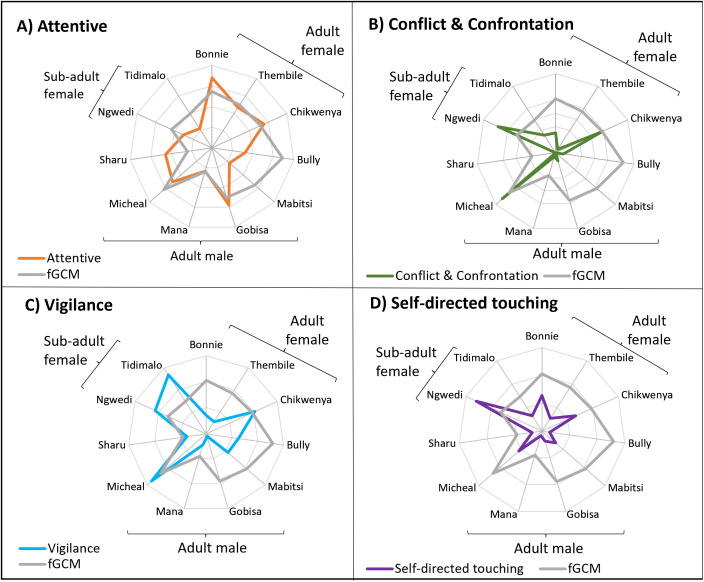
Radar graph illustrating the relationship between A) Attentive, B) Conflict & Confrontation, C) Vigilance, and D) Self-directed touching and the faecal glucocorticoid metabolite (fGCM) concentration (μg/g DW) exhibited by the rewilded elephants.

Even though the relationship between the mean behavioural frequencies and mean fGCM concentrations observed for the rewilded elephants could not be statistically analysed, Fig 8 illustrates varying strengths of relationships between these two parameters. The mean frequency of each behavioural category showed a unique relationship with the mean fGCM concentrations reported for each rewilded elephant. All the rewilded AFs, Michael (AM) and Gobisa (AM), exhibited a high correspondence between Attentive behavioural frequencies and fGCM concentrations. Only Chikwenya (AF) and Michael (AM) showed a strong relationship between the Conflict and Confrontation and Vigilance behavioural frequencies and fGCM concentrations. Mana (AM) and Sharu (AM) exhibited low frequencies of behaviours within the Conflict & Confrontation, Vigilance, and Self-directed touching categories, as well as low mean fGCM concentrations. In contrast, Bonnie (AF), Thembile (AF) Mabitsi (AM), Bully (AM), and Gobisa (AM) exhibited low behavioural frequencies within all categories (except Attentive); however, these low frequencies corresponded with relatively high mean fGCM concentrations.

## Discussion

A variety of practical tools have been developed to assess the well-being of elephants in captivity [14,70–74] and in the wild [37,42,75]. Despite the success in assessing the well-being of elephants in these two environments (wild or captive), few opportunities have arisen to implement a quantitative assessment of the different behavioural and physiological responses rewilded elephants (ex-captive) show in a wild environment. Reporting on the ability of rewilded elephants to adapt and respond to natural stimuli is a key area of research to provide answers to those still critical of reintegration as an option available to captive elephants. The study can also provide the necessary scientific, ethos and information to generate a realistic expectation for stakeholders wanting to secure land for these captive elephants to rewild them. Preliminary insight into a potential range of behavioural and physiological responses of rewilded elephants can also be gained from this research. These insights are highly dependent on the group dynamics during their reintegration and after they have been rewilded, their captive histories, and what reintegration process they were exposed to. As such, the findings should inform, rather than define, expectations regarding the adaptation and well-being of rewilded elephants in future conservation or management scenarios. In our study, each elephant’s focal and faecal samples were collected across various natural scenarios to generate an overall behavioural profile and insight into their physiological responses. Previous studies have shown that animal behaviour can be situationally dependent but should not vary too much during similar long-term environmental conditions, such as elephant social structure, presence of predators, and other large herbivores, etc. [76,77]. Therefore, the results should be interpreted cautiously as some situations could have potentially caused spikes in specific behavioural frequencies and physiological responses.

### Behavioural differences between wild and rewilded elephants

It was hypothesised that rewilded elephants would exhibit higher frequencies of Conflict and Confrontation and Self-directed touching (SDT) behaviours due to these often being prevalent in captive scenarios [14,17,18,59,78]. Higher frequencies of Attentive and Vigilant behaviour were also expected for rewilded elephants, as they might be less habituated to wild pressures than their wild counterparts. Contrary to our expectation, the data did not support these hypotheses due to grouped rewilded adult females (AF), adult males (AM), and sub-adult females (SAF) showing similar frequencies of all behavioural categories, except Attentive behaviour, to those reported for their wild counterparts. These behavioural similarities between rewilded and wild elephants within these three behavioural categories should be considered as positive, as they may indicate that the rewilded study elephants have been able to adopt normal elephant behaviour through their years spent as free-roaming elephants. However, it is difficult to deduce whether their behaviour has changed or improved over the years of roaming free, but the similarities to wild elephants do indicate that they are not showing signs of chronic stress, often associated with behavioural abnormalities [79] and stereotypes [12]. The results observed for the Attentive behavioural category were inconsistent with what was hypothesised, as the wild AMs and wild SAFs showed significantly higher frequencies of Attentive behaviours than the rewilded AMs and SAFs. Attentive behaviour includes signals such as ‘freezing’ to look or listen, ‘smelling’, ‘exploring objects’, etc., which elephants exhibit when they want to gain more information regarding their direct environment, such as identifying potential threats such as predators [80–82]. This may be attributed to the fact that in captivity, males and females are normally managed as one group and are always under the supervision and guidance of handlers, and therefore, perhaps they have had less of a need to assess their surroundings due to dependence on handlers. The rewilded SAFs were juveniles whilst in captivity and they did not take on the role of the responsible elephant in their captive environment, whereas, in the wild, sub-adult females may need to be more attentive from an earlier age to respond to natural stressors such as caring for their young, predation, sourcing fodder and water, disease, and injury [83]. Alternatively, Ngwedi’s (SAF) and Tidimalo’s (SAF) lower frequencies of Attentive behaviour could be balanced by Chikwenya (AF), their Matriarch’s elevated attentiveness and vigilance.

Interestingly, some rewilded elephants within the same reserve showed significantly different behavioural frequencies within some of the categories. The latter could be attributed to personality differences. The personality of an animal can be described as inter-individual behavioural differences that remain consistent over time and across contexts [77,84]. In principle, we would expect that individuals within the same age and sex group, who were exposed to the same captive history, reintegration program, and reserve characteristics, exhibit similar behaviours. However, literature has shown that individuals adjust their behaviour according to current conditions [85], which may be perceived differently by certain personality types. Different elephants may have obtained diverse coping styles [85,86] to respond during various circumstances, resulting in behavioural differences.

Furthermore, no significant differences were reported between the wild and grouped rewilded AFs, when assessing the frequencies of Attentive behaviour. One rewilded AF (Chikwenya), did, however, show significantly higher frequencies of Attentive behaviour than the Wild AFs. Even though not significant, she also exhibited the highest mean frequencies within all the behavioural categories. She has spent the longest time in captivity (± 27 years) and has been roaming free for the shortest period (4–9 years) in comparison to the other two AFs (± 17 years in captivity; 10–14 years free) in the study. It is important to consider that Chikwenya (AF) is the oldest female on the reserve and was released as the only adult female responsible for all the offspring present throughout the study period, whereas the wild AFs and the other two rewilded AFs (Bonnie and Thembile) in the study all had other adult females to consolidate with. It has also been shown that social buffering (supportive, conspecific relationships) can lead to decreased stress responses due to shared responsibility and decision-making [26,30,87,88].

### *Faecal glucocorticoid metabolite* (*fGCM*) concentrations

We would expect to see transitory increases in fGCM levels when stressors are present over a prolonged period [49], when there is conflict within the herd, anthropogenic or natural disturbances are present, etc. [44], and eventually these levels will decline again when homeostasis is established. Early life trauma or long-term stress can ultimately result in structural remodelling of the brain, which in turn affects the way that an individual responds on a physiological level [89]. These changes can alter the GC regulation, which in turn affects the GC response, whether it is the baseline concentrations, stress-induced concentrations, or the negative feedback system [50]. In a captive system, long-term stress can be caused by restricted freedom of choice and fewer opportunities to avoid certain situations, such as training or tourist interactions [11]. In the wild, the most common long-term stressors are scarcity of food and water availability, as well as challenging social interactions [50,83]. When evaluating well-being in elephants, it is important to understand the difference between perceived stress and distress. The term stress can be divided into non-threatening stimuli, known as ‘good’ stress (eustress or during pregnancy and parturition), whereas ‘bad’ stress refers to stimuli that can cause harm to the animal (e.g., predators) [90]. Being in a wild environment, the rewilded elephants are exposed to different stressors at high frequencies, not to mention wildlife management interventions and tourism activities, which could elicit fluctuations in physiological responses as well [37,42].

We hypothesised that rewilded elephants would have higher fGCM concentrations (higher HPA axis activity) than their wild counterparts due to their past life experiences [23] and potentially being less habituated to natural pressures associated with free-living. The fGCM concentrations obtained from the AFs and SAFs supported this hypothesis, as significantly higher fGCM concentrations were reported for rewilded elephants than those noted for wild elephants. Garai et al. 2022 reported similar results where rewilded elephants on one of the research reserves had significantly higher fGCM concentrations than wild elephants. These differences between the rewilded and wild AFs and SAFs, can potentially point towards different personalities [24] or different energy requirements during different periods, such as long-distance movement, reproduction status (pregnancy or calf rearing) [91], seasonal effects [92], etc. One study has reported a positive correlation between the fGCM concentrations of wild Asian elephants and the number of calves that were present [93]. Throughout our study period, the wild AFs and SAFs (Reserve 4) were roaming in herds where calves were present, which was also the case for Chikwenya (AF), Ngwedi (SAF), and Tidimalo (SAF) in Reserve 2. Although Bonnie (AF) and Thembile (AF) were not reported to be pregnant or have small calves during the study period, their fGCM concentrations were still higher than those of the wild AF. The rewilded AFs also had varying captive histories, time spent in captivity, and time spent roaming free, yet when the influence of these factors was tested, no significant differences were reported for the AFs that spent 15–17 years (Bonnie and Thembile) and 24–27 years in captivity (Chikwenya). The latter may point towards other factors potentially having a larger influence on the fGCM concentrations within the AF age class.

There were no significant differences found when comparing the fGCM concentrations of the rewilded AM group with their wild counterparts. However, when the individual rewilded AMs were considered, Bully (AM), Mabitsi (AM), and Michael (AM) did have significantly higher fGCM concentrations than the wild AMs. Bully (AM), who had the highest fGCM concentrations of all the males, was raised in a circus, which is associated with confined space, lack of stimulation, and perhaps fewer opportunities to experience and learn to cope with novel situations [94–96]. Bully has also been roaming free for the longest period (12–14 years) and did not spend the longest time (15 years) in captivity in comparison to the other AMs (4–14 years free; 24–27 years in captivity). This potentially indicates that time spent in captivity and years roaming free did not have the biggest effect on the fGCM concentrations of the AMs. Both Bully and Mabitsi (AM) are housed in the same reserve (Reserve 1), which could also suggest that certain triggers in their current environment could contribute to the high fGCM concentrations, such as an absence of other bulls to socialise with, a lack of sufficient food sources (housed in Fynbos habitat), and being vaccinated with GnRH from a helicopter biannually, and exposure to high tourism viewing activities. Another bull with high fGCM levels was Michael (AM), who roams on the same 90,000 ha reserve as Mana (AM) and Sharu (AM) (Reserve 2). This highlights the variation in fGCM concentrations between different individuals, as well as inter-individual variation [97], reaffirming that we cannot expect to observe similar concentrations for individuals due to similar captive histories or current living conditions. Ganswindt et al. (2010) reported a median fGCM concentration of 0.78 µg/g DW with a range of 0.5–1.2 µg/g DW for an adult bull during periods of being physically injured (stressful period); however, in our study, none of the rewilded AMs had a median fGCM concentration above 0.514 µg/g DW. This may indicate that none of the rewilded bulls experienced prolonged intervals of pressure or significant stressors during the study period; however, peaks in fGCM concentrations could have been caused by various intrinsic (reproductive (musth) status or injury) [47,56] and extrinsic factors (drought, competition, predation, etc.) [42,54,98]. Rainfall season only had a significant effect on the fGCM concentrations of rewilded AMs, where higher concentrations coincided with the wet season. This was unexpected, as literature has shown that higher concentrations are normally associated with the dry season [42,44,54]. For many of these small reserves, the wet season often occurs during the same period considered as the high tourism season, which has been shown to have a significant effect on the physiological responses of elephants [42,53]. Furthermore, it needs to be highlighted that whilst some of the rewilded AFs, AMs and SAFs had significantly higher fGCM concentrations than their wild counterparts, their baseline (median) values were comparable to those concentrations reported in the literature [14,37,42,47,99].

### Relationship between behavioural and physiological responses of the rewilded elephants

When assessing whether a relationship exists between the mean behavioural frequencies and mean fGCM concentrations exhibited by the rewilded elephants, most elephants (except Bully (AM) and Mabitsi (AM)) showed a strong correlation between mean Attentive behaviour and mean fGCM concentrations. Interestingly, Michael (AM) and Chikwenya (AF) both showed high correspondence between mean behavioural frequencies and fGCM concentrations for all the behavioural categories. Both Mana (AM) and Sharu (AM) showed low frequencies within most of the categories, which correlated with relatively low fGCM concentrations. This shows that for certain individuals, behaviour could be an accurate predictor of certain physiological responses and vice versa; for example, a rewilded elephant exhibiting low mean behavioural frequencies might be expected to have lower fGCM concentrations, and the same goes for elephants showing higher behavioural frequencies, where higher fGCM concentrations may be expected. Garaï et al. (2022) reported a similar trend where rewilded elephants that showed high behavioural frequencies also exhibited high fGCM concentrations. However, this was not the case with many of the rewilded elephants in our study, and some elephants even showed opposite trends for behavioural and physiological responses. Therefore, the hypothesis stating that there is a relationship between behavioural and physiological responses can only be partially confirmed. Because of these behavioural and physiological inconsistencies, further research is required involving long-term monitoring of individual elephants to improve our understanding of these underlying differences.

One of the primary limitations of this study was the scarce availability of rewilded elephants in South Africa, resulting in a very small sample size. Even so, approximately 20 ex-captive elephants have been rewilded in South Africa, which means that the study animals do represent more than 50% of the rewilded elephant population across the country. To ensure that enough focal and faecal samples were collected for each elephant over a long period, different observation periods were conducted at each reserve. Due to limitations regarding accessibility of elephants during each period, evenly spread data across the different periods could not be ensured, and therefore, some periods were associated with a lot of samples per elephant, whereas during others, no samples were collected from certain elephants. Thus, the findings offer only preliminary insights, serving as an overview of behavioural and physiological responses across many years. Another limitation was that we did not have enough data on individual wild elephants, and they therefore had to be grouped into the three age and sex categories (AF, AM, SAF). The latter was due to APNR being an open system with a large population size (± 3607), which poses a challenge when aiming to encounter and collect various samples for key individuals for the study. Lastly, there were not always wild elephants within the different age/ sex categories to compare the rewilded elephants to. In reserves where there were other wild elephants, we could not gather enough data on wild elephants to compare the behaviour and physiological responses of the rewilded elephants to, therefore, it was decided to compare all rewilded elephants to the same baseline wild population.

## Conclusion

This study demonstrated that rewilded elephants expressed similar behavioural frequencies to their wild counterparts, except within the Attentive category, where both rewilded AMs and SAFs showed lower frequencies than wild elephants. Even after spending decades in captivity, individual rewilded elephants have established unique behavioural and physiological response mechanisms to process and react to environmental stimuli, whether it is actively or passively. We tentatively attributed these distinctive differences mainly to personality traits that can result in some individuals appearing more responsive to their environment, while others are perceived as less responsive. All rewilded AFs and SAFs, and some individual rewilded AMs had comparatively higher fGCM concentrations than the wild elephants; however, all these values fell within the normal ranges of wild elephants, which was considered a positive observation. When assessing whether a relationship exists between behavioural and physiological responses, it was suggested that Attentive behaviour had a strong correlation with fGCM concentrations of the rewilded elephants. Many of the rewilded elephants did show a strong positive and negative relationship between behavioural and physiological responses; however, some did not. Therefore, these interpretations need to be made with caution.

## Supporting information

S1 Table The mean frequencies (per 10-min) ± SD of the detailed behaviours that were exhibited more than 4% of the total frequencies of behaviours expressed within each of the three groups (AF, AM, and SAF) that were analysed. N = number of focal samples (some detailed behaviours were not included for AMs and SAFs as they did not meet our 4% threshold).
(DOCX)

S2 Table Mean (M) ± SD faecal glucocorticoid metabolite (fGCM) concentrations (number of samples), as well as the coefficient of variance (CV %) of the rewilded elephants within the four reserves during the different data collection periods throughout the study. Grey blocks indicate periods where at least one of these musth signs was present (urine dribbling, penis decolouration, temporal gland secretion, or swollen temporal gland).
(DOCX)

S1 Fig Box plot illustrating the faecal glucocorticoid metabolite concentration (μg/g DW) of rewilded and wild elephants in the study reserves. The red crosses represent means, whereas the central horizontal bars show medians. The first and third quartiles are the lower and upper limits of the box, respectively. The length of the whiskers indicates 1.5 times the interquartile range. The grey points represent the minimum and maximum values (N = number of faecal samples).
(DOCX)

S1 File Elephant behaviour and fGCM data used in the manuscript. (XLSX)
