# Supplementary material for: Investigating the adaptive coping mechanisms of rewilded elephants: A comparison of behavioural and physiological variables with wild elephants
Source: PLoS One. 2026 Jul 29;21(7):e0348698. doi: 10.1371/journal.pone.0348698 (PMC13419208; doi:10.1371/journal.pone.0348698)
Supplement: S1 Table — N = number of focal samples (some detailed behaviours were not included for AMs and SAFs as they did not meet our 4% threshold). (DOCX) [file pone.0348698.s001.docx]

***S1 Table*** The mean frequencies (per 10-min) ± SD of the detailed behaviours that were exhibited more than 4% of the total frequencies of behaviours expressed within each of the three groups (AF, AM, and SAF) that were analysed. N= number of focal samples (some detailed behaviours were not included for AMs and SAFs as they did not meet our 4% threshold).

| **Detailed Behaviours** | **Adult females** | | | **Adult males** | | | **Sub-adult females** | | |
| --- | --- | --- | --- | --- | --- | --- | --- | --- | --- |
|  | Wild (N=20) | Rewilded (N=60) | p-value | Wild (N=20) | Rewilded  (N=117) | p-value | Wild (N=39) | Rewilded  (N=19) | p-value |
| LTS-AIR | 0.050  ± 0.224 | 0.633  ± 1.473 | 0.895 | 0.150 ± 0.489 | 0.325  ± 0.927 | 0.895 |  | N/A |  |
| SMA | 2.000  ± 2.492 | 3.550  ± 4.106 | 0.102 | 4.150  ± 3.573 | 2.855  ± 4.320 | 0.102 | 4,632  ± 3,685 | 1,769  ± 2,182 | 0.102 |
| SPL | 0.900  ± 1.071 | 1.367  ± 2.484 | **0.038** | 2.000  ± 2.492 | 0.564  ± 1.078 | **0.038** |  | N/A |  |
| FSW | 0.200  ± 0.523 | 0.717  ± 1.059 | 0.401 | 0.700  ± 1.031 | 0.726  ± 1.418 | 0.401 | 1,000  ± 1,795 | 1,59  ± 2,197 | 0.401 |
| REAR | 0.950  ± 1.877 | 0.333  ± 0.729 | **< 0.001** |  | N/A |  | 1,263  ± 2,978 | 0,333  ± 0,737 | **< 0.001** |
| TEF | 0.250  ± 0.444 | 1.417  ± 3.066 | 0.406 | 0.400  ± 0.821 | 0.444  ± 1.012 | 0.406 | 1,211  ± 2,347 | 1,154  ± 1,725 | 0.406 |
| TMO | 0.850  ± 1.424 | 1.550  ± 1.692 | 0.476 | 0.900  ± 1.294 | 0.966  ± 2.042 | 0.476 | 2,947  ± 2,953 | 2,718  ± 3,873 | 0.476 |
| TLF | 0.200  ± 0.523 | 0.550  ± 1.241 | 0.143 |  | N/A |  | 1,842  ± 4,059 | 2,333  ± 6,127 | 0.143 |
| TTT | 0.050  ± 0.224 | 0.517  ± 1.513 | 0.207 |  | N/A |  | 0,474  ± 1,073 | 0,641  ± 2,032 | 0.207 |
