# Supplementary material for: Investigating the adaptive coping mechanisms of rewilded elephants: A comparison of behavioural and physiological variables with wild elephants
Source: PLoS One. 2026 Jul 29;21(7):e0348698. doi: 10.1371/journal.pone.0348698 (PMC13419208; doi:10.1371/journal.pone.0348698)
Supplement: S2 Table — Grey blocks indicate periods where at least one of these musth signs was present (urine dribbling, penis decolouration, temporal gland secretion, or swollen temporal gland). (DOCX) [file pone.0348698.s002.docx]

***S2 Table*** Mean (M) ± SD faecal glucocorticoid metabolite (fGCM) concentrations (number of samples)*, as well as the coefficient of variance (CV %)* of the rewilded elephants within the four reserves during the different data collection periods throughout the study. Grey blocks indicate periods where at least one of these musth signs was present (urine dribbling, penis decolouration, temporal gland secretion, or swollen temporal gland).

| ***Reserve 1*** | ***Elephant*** |  | ***Dry*** | | | ***Wet*** | | | |  | **Mean *± SD*** |
| --- | --- | --- | --- | --- | --- | --- | --- | --- | --- | --- | --- |
|  | ***ID*** |  | **Jan 2020** | **Jan-Feb 2021** | **Mar-May 2024** | **Jun-Jul 2020** | **Sept-Oct 2021** | **Aug-Oct 2023** | **Jun-Jul 2024** |  |  |
|  | ***Bonnie (AF)*** | *M±SD* | *0.41 ± 0.06 (3)* | *-* | *0.34 ± 0.05 (2)* | *0.44 (1)* | *0.44 ± 0.06 (2)* | *0.41 ± 0.04 (3)* | *0.45 (1)* |  | *0.41 ± 0.06 (12)* |
|  |  | *CV %* | *14.66* |  | *13.05* | *N/A* | *14.62* | *9.42* | *N/A* |  | *14.20* |
|  | ***Thembile (AF)*** | *M±SD* | *-* | *-* | *-* | *0.32 (1)* | *0.38 (1)* | *0.35 (1)* | *0.43 (1)* |  | *0.37 ± 0.04 (4)* |
|  |  | *CV %* |  |  |  | *N/A* | *N/A* | *N/A* | *N/A* |  | *10.86* |
|  | ***Bully***  ***(AM)*** | *M±SD* | *0.25 (1)* | *-* | *0.33 ± 0.06 (2)* | *0.54 ± 0.13 (3)* | *0.46 ± 0.03 (2)* | *0.66 ± 0.11 (5)* | *-* |  | *0.52 ± 0.17 (13)* |
|  |  | *CV %* | *N/A* |  | *19.52* | *24.99* | *5.67* | *16.08* |  |  | *32.57* |
|  | ***Mabitsi (AM)*** | *M±SD* | *0.44 ± 0.09 (4)* | *-* | *0.34 ± 0.07 (4)* | *0.36 ± 0.08 (3)* | *0.23 (1)* | *0.44 ± 0.19 (5)* | *0.54 ± 0.01 (3)* |  | *0.41 ± 0.13 (20)* |
|  |  | *CV %* | *20.58* |  | *19.55* | *20.57* | *N/A* | *42.27* | *2.67* |  | *31.71* |
|  | ***Total*** |  | ***8*** | ***-*** | ***8*** | ***8*** | ***6*** | ***14*** | ***5*** |  | ***49*** |
| ***Reserve 2*** | ***Elephant*** |  | ***Dry*** | | | | | ***Wet*** | | | **Mean*± SD*** |
|  | ***ID*** |  | **May-June 2019** | **Aug-Oct 2020** | **May 2022** | **Jul 2023** | **Apr-May 2024** | **Jan 2021** | **Nov 2021** | **Feb 2023** |  |
|  | ***Chikwenya (AF)*** | *M±SD* | *0.62 (1)* | *0.42 ± 0.09 (4)* | *0.33 ± 0.07 (4)* | *0.28 ± 0.05 (3)* | *-* | *0.41 ± 0.14 (8)* | *0.39 ± 0.03 (2)* | *-* | *0.39 ± 0.13 (22)* |
|  |  | *CV %* | *N/A* | *22.20* | *21.12* | *18.91* |  | *34.37* | *8.00* |  | *14.20* |
|  | ***Michael (AM)*** | *M±SD* | *0.36 ± 0.11 (6)* | *0.43 ± 0.08 (14)* | *0.62 ± 0.05 (3)* | *-* | *-* | *0.52 ± 0.15 (7)* | *-* | *0.59 ± 0.10 (2)* | *0.47 ± 0.13 (32)* |
|  |  | *CV %* | *30.36* | *19.36* | *7.43* |  |  | *29.00* |  | *17.15* | *28.64* |
|  | ***Mana (AM)*** | *M±SD* | *0.11 ± 0.04 (14)* | *0.14 ± 0.14 (8)* | *0.49 ± 0.16 (3)* | *-* | *0.27 ± 0.04 (4)* | *0.21 ± 0.05 (6)* | *-* | *0.10 ± 0.02 (3)* | *0.18 ± 0.14 (38)* |
|  |  | *CV %* | *37.92* | *95.64* | *32.63* |  | *16.00* | *22.34* |  | *22.97* | *76.57* |
|  | ***Sharu***  ***(AM)*** | *M±SD* | *0.10 ± 0.04 (6)* | *0.54 (1)* | *0.23 ± 0.08 (4)* | *0.10 ± 0.01 (2)* | *0.13 ± 0.03 (2)* | *0.11 ± 0.02 (2)* | *0.29 ± 0.06 (3)* | *0.22 (1)* | *0.18 ± 0.12 (21)* |
|  |  | *CV %* | *35.83* | *N/A* | *34.59* | *14.67* | *22.53* | *13.46* | *20.95* | *N/A* | *65.19* |
|  | ***Tidimalo (SAF)*** | *M±SD* | *0.37 (1)* | *0.30 ± 0.08 (9)* | *0.34 (1)* | *0.22 (1)* | *-* | *0.26 ± 0.03 (4)* | *0.33 ± 0.02 (2)* | *-* | *0.29 ± 0.07 (18)* |
|  |  | *CV %* | *N/A* | *25.38* | *N/A* | *N/A* |  | *11.74* | *4.77* |  | *21.96* |
|  | ***Ngwedi (SAF)*** | *M±SD* | *-* | *0.31 ± 0.06 (3)* | *0.37 ± 0.05 (2)* | *0.25 ± 0.03 (2)* | *-* | *0.34 ± 0.04 (5)* | *0.40 (1)* | *0.26 (1)* | *0.32 ± 0.06 (14)* |
|  |  | *CV %* |  | *18.07* | *12.50* | *10.49* |  | *13.13* | *N/A* | *N/A* | *18.74* |
|  | ***Total*** |  | ***28*** | ***39*** | ***17*** | ***8*** | ***6*** | ***32*** | ***8*** | ***7*** | ***145*** |
| ***Reserve 3*** | ***Elephant*** |  | ***Dry*** | | | ***Wet*** | | |  |  | **Mean *± SD*** |
|  | ***ID*** |  | ***Aug 2019*** | ***Sept 2022*** | ***July 2023*** | ***Dec 2020*** | ***Oct 2021*** | ***Feb 2024*** |  |  |  |
|  | ***Gobisa (AM)*** | *M±SD* | *0.35 ± 0.09 (2)* | *0.54 ± 0.15 (2)* | *0.37 ± 0.05 (3)* | *0.18 (1)* | *0.35 ± 0.04 (2)* | *0.38 ± 0.04 (3)* |  |  | *0.38 ± 0.12 (13)* |
|  |  | *CV %* | *25.94* | *28.62* | *12.70* | *N/A* | *12.59* | *11.49* |  |  | *30.62* |
|  | ***Total*** |  | ***2*** | ***2*** | ***3*** | ***1*** | ***2*** | ***3*** |  |  | ***13*** |
| ***Reserve 4*** | ***Elephant*** |  | ***Dry*** | | ***Wet*** | | |  |  |  | **Mean *± SD*** |
|  | ***ID*** |  | **Apr 2019** | ***Aug-Sept 2021*** | ***Nov-Dec 2019*** | ***Nov 2020*** | ***Mar-Apr 2022*** |  |  |  |  |
|  | ***Wild AF*** | *M±SD* | *0.21 ± 0.17 (3)* | *-* | *0.26 ± 0.12 (9)* | *0.29 ± 0.16 (12)* | *-* |  |  |  | *0.28 ± 0.15 (24)* |
|  |  | *CV %* | *58.61* |  | *44.49* | *55.61* |  |  |  |  | *52.97* |
|  | ***Wild AM*** | *M±SD* | *0.22 ± 0.15 (8)* | *-* | *0.31 ± 0.15 (7)* | *0.26 ± 0.01 (3)* | *-* |  |  |  | *0.26 ± 0.14 (18)* |
|  |  | *CV %* | *68.94* |  | *48.01* | *4.12* |  |  |  |  | *54.57* |
|  | ***Wild SAF*** | *M±SD* | *0.18 ± 0.04 (4)* | *0.23 ± 0.01 (2)* | *0.20 ± 0.03 (2)* | *0.19± 0.01 (4)* | *0.25 ± 0.12 (3)* |  |  |  | *0.20 ± 0.06 (15)* |
|  |  | *CV %* | *22.92* | *5.67* | *14.89* | *7.75* | *47.55* |  |  |  | *31.35* |
|  |  | ***Total*** | ***15*** | ***2*** | ***18*** | ***19*** | ***3*** |  |  |  | ***57*** |
