# Supplementary material for: Investigating the adaptive coping mechanisms of rewilded elephants: A comparison of behavioural and physiological variables with wild elephants
Source: PLoS One. 2026 Jul 29;21(7):e0348698. doi: 10.1371/journal.pone.0348698 (PMC13419208; doi:10.1371/journal.pone.0348698)
Supplement: S1 Fig — The red crosses represent means, whereas the central horizontal bars show medians. The first and third quartiles are the lower and upper limits of the box, respectively. The length of the whiskers indicates 1.5 times the interquartile range. The grey points represent the minimum and maximum values (N = number of faecal samples). (DOCX) [file pone.0348698.s003.docx]

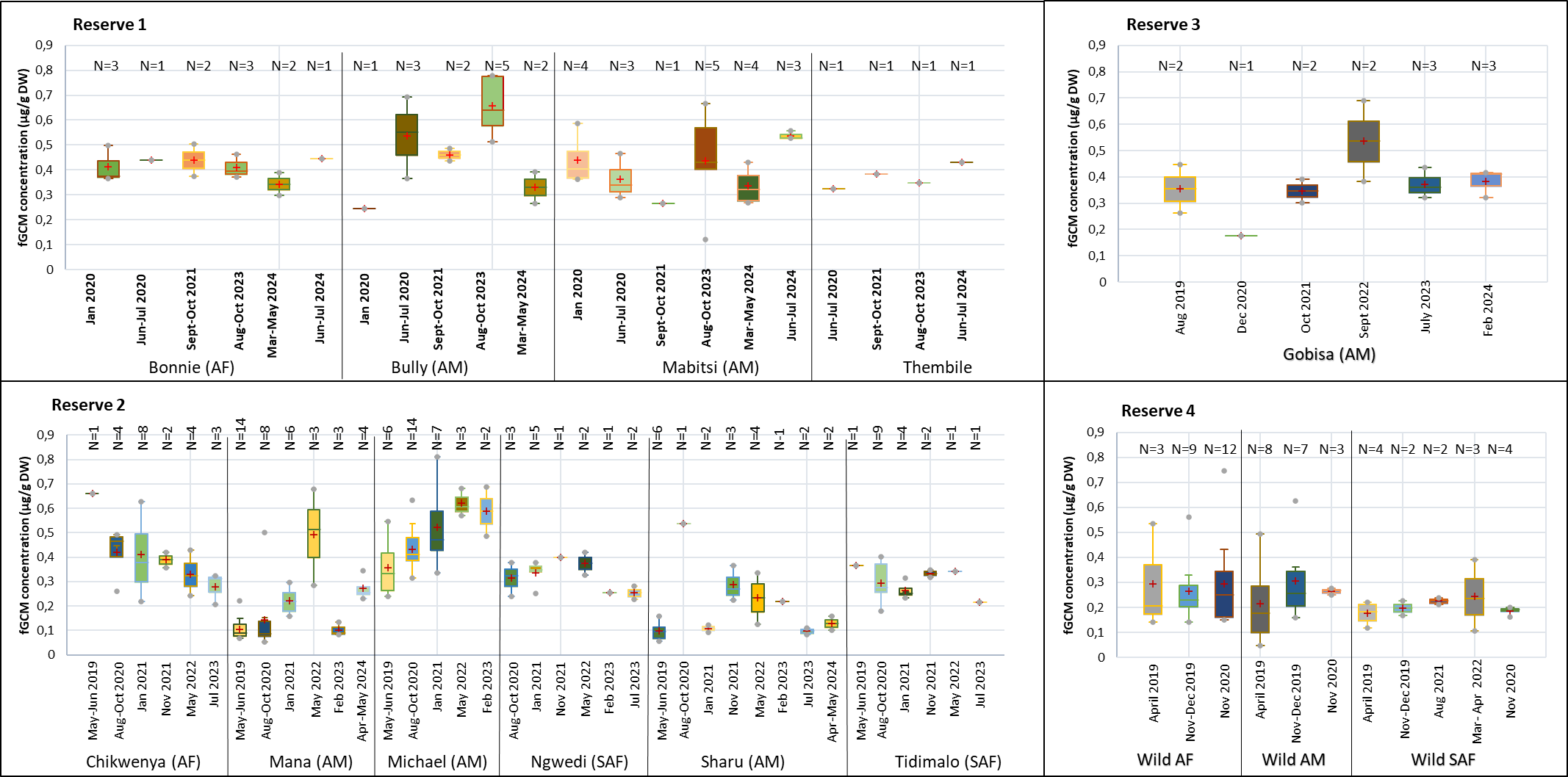


***Figure S1*** Box plot illustrating the faecal glucocorticoid metabolite concentration (μg/g DW) of rewilded and wild elephants in the study reserves. The red crosses represent means, whereas the central horizontal bars show medians. The first and third quartiles are the lower and upper limits of the box, respectively. The length of the whiskers indicates 1.5 times the interquartile range. The grey points represent the minimum and maximum values (N=number of faecal samples).
